# Apolipoprotein L1 risk genotypes and odds of lupus nephritis-associated kidney failure in systemic lupus erythematosus: a systematic review and meta-analysis

**DOI:** 10.3389/fmed.2026.1903888

**Published:** 2026-08-19

**Authors:** Silvia E. Aldana-Pérez, Alex Dominguez-Vargas, Gustavo Aroca-Martinez, Ana Moreno-Woo, Luis Fang, Gloria Garavito De Egea, Eduardo Egea Bermejo, Henry J. González-Torres

**Affiliations:** 1 Centro de Investigaciones en Ciencias de la Vida, Facultad de Ciencias de la Salud, Universidad Simón Bolívar, Barranquilla, Colombia; 2 Data Analysis and Mining Department, D&P Consulting Service SAS, Barranquilla, Colombia; 3 División Ciencias de la Salud, Universidad del Norte, Barranquilla, Colombia; 4 Departamento de Medicina Interna, Clínica de la Costa, Barranquilla, Colombia

**Keywords:** *APOL1*, end-stage renal disease, genetic association, lupus nephritis, renal prognosis

## Abstract

**Background:**

Systemic lupus erythematosus (SLE) is characterized by a prominent interferon signature that upregulates apolipoprotein L1 (*APOL1*) expression. High-risk *APOL1* genotypes have been associated with end-stage renal disease (ESRD) as a complication of lupus nephritis (LN); however, the strength and consistency of this association remain heterogeneous across studies.

**Methods:**

We conducted a systematic review and meta-analysis of observational studies to evaluate the association between *APOL1* genotypes and the odds of LN-associated kidney failure among patients with SLE. A systematic search of PubMed, Scopus, and Web of Science identified eligible observational studies, and pooled odds ratios with 95% confidence intervals were estimated using random-effects models. Between-study heterogeneity and publication bias were assessed using standard methods.

**Results:**

Four observational studies comprising a total of 1,885 patients of African ancestry were included. Of these, 321 carried high-risk *APOL1* genotypes and 1,564 carried low-risk genotypes. High-risk *APOL1* genotypes were associated with significantly increased odds of LN-associated ESRD compared with low-risk genotypes (OR 2.66; 95% CI 1.63–4.32; *p* = 0.008), with low heterogeneity (*I*^2^ = 9.8%). Chronic kidney disease outcomes were reported heterogeneously and were summarized descriptively.

**Conclusion:**

High-risk *APOL1* genotypes are associated with significantly increased pooled odds of LN-associated ESRD among patients with SLE. These findings demonstrate that *APOL1* is a clinically relevant genetic modifier of renal prognosis, supporting its potential role as a tool for risk stratification and precision medicine approaches in LN.

**Systematic review registration:**

https://www.crd.york.ac.uk/PROSPERO/view/CRD42024587943, identifier PROSPERO (CRD42024587943).

## Introduction

Systemic lupus erythematosus (SLE) is a complex, multifactorial autoimmune disease characterized by the production of high titers of autoantibodies against nuclear antigens (1). The disease disproportionately affects individuals of African ancestry compared with those of European ancestry and is associated with a more severe clinical course, a disparity likely driven by a complex interplay of genetic, socioeconomic, and environmental factors (2, 3). Patients of African ancestry exhibit higher rates of lupus nephritis (LN) and a nearly threefold increased risk of progression to end-stage kidney disease (ESKD) compared with White patients (4, 5).

Current evidence suggests that this excess risk may be partially mediated by two kidney risk variants (G1 and G2) in the apolipoprotein L1 (*APOL1*) gene (6). Unlike most common genetic variants associated with complex diseases, APOL1-mediated kidney disease follows a predominantly autosomal-recessive pattern of inheritance (7). The *APOL1* gene, located on chromosome 22, encodes apolipoprotein L1, a member of the APOL family implicated in innate immune responses (6). Importantly, *APOL1* transcription is upregulated by inflammatory stimuli, particularly those mediated through interferon-dependent pathways (8). Given that SLE is characterized by chronic activation of the interferon system, with increased expression of interferon-regulated genes (8, 9), this biological context provides a mechanistic basis for the observed association between *APOL1* high-risk (HR) genotypes, defined as the presence of two risk alleles (G1/G1, G1/G2, or G2/G2), and the development of ESKD as a complication of LN (10, 11).

Despite this biologically plausible link, the strength of the association between *APOL1* HR genotypes and adverse renal outcomes has been highly heterogeneous across studies (6, 12). While several investigations report a strong association between *APOL1* HR status and kidney disease, others describe more modest or inconsistent effects. Reported odds ratios for chronic kidney disease range from 1.5 to 2.0 in otherwise healthy individuals, increase to approximately 2.5–14 in patients with LN, and reach 29–80 in the context of HIV-associated nephropathy (12–14).

Although individual observational studies and narrative reviews have examined the role of *APOL1* risk variants in SLE and LN (10, 11), no prior systematic review or meta-analysis has quantitatively synthesized the available evidence to estimate the magnitude of the association between *APOL1* genotype and renal outcomes, particularly ESKD, in patients with LN. Therefore, we conducted a systematic review and meta-analysis of observational studies to evaluate the association between *APOL1* risk genotypes and the odds of chronic kidney disease (CKD) and LN-associated ESKD among patients with SLE.

## Methods

### Search strategy and study selection

This systematic review and meta-analysis were conducted in accordance with the Preferred Reporting Items for Systematic Reviews and Meta-Analyses (PRISMA 2020) guidelines. The study protocol was prospectively registered in the International Prospective Register of Systematic Reviews (PROSPERO) under registration number CRD42024587943. A systematic search of PubMed, Scopus, and Web of Science was performed up to July 2026. Only articles published in English were considered. The search strategy combined database-specific controlled vocabularies, including MeSH terms for PubMed, and free-text keywords related to SLE and *APOL1*. The complete search strategy, which was adapted for the other databases, used the following combinations: *(“Lupus” OR “Systemic Lupus Erythematosus” OR “SLE” OR “Lupus nephritis”) AND (“APOL1” OR “apolipoprotein L1”).* In addition, the reference lists of all eligible articles and relevant reviews were manually screened to identify additional studies not captured by the electronic search.

### Eligibility criteria and data extraction

Study eligibility was defined according to the PICOTT (Population, Intervention, Comparator, Outcome, Type of study, Time) framework (15). Studies were included if they met the following criteria: (1) observational study design, including cohort or case–control studies; (2) inclusion of patients diagnosed with SLE according to standardized and validated international classification criteria (e.g., the American College of Rheumatology [ACR] criteria or the Systemic Lupus International Collaborating Clinics [SLICC] criteria), with outcome assessment for LN and ESKD; (3) assessment of *APOL1* genotypes, with classification into HR and low-risk (LR) categories; (4) reporting of LN-associated ESKD among patients with SLE as the primary outcome (with or without additional reporting of CKD outcomes) and (5) provision of effect estimates, such as odds ratios, risk ratios, or hazard ratios, or sufficient raw data to calculate them. Studies were excluded if they were animal studies, reviews, meta-analyses, editorials, conference abstracts, or lacked sufficient data for quantitative synthesis. In cases of duplicate or overlapping populations, the most informative or recent publication was retained.

Two investigators independently screened titles and abstracts, reviewed full texts for eligibility, and extracted data using a standardized collection form. The extracted information included the first author, year of publication, study design, population characteristics such as sample size, age, sex, and ancestry, as well as follow-up duration, *APOL1* genotype classification, renal outcomes, and raw 2 × 2 contingency data (event counts and total sample sizes per genotype cohort) required for unadjusted quantitative synthesis. Any discrepancies during the process were resolved by consensus or through consultation with a third reviewer.

### Quality assessment

The methodological quality of the included studies was independently assessed by two reviewers using the Newcastle–Ottawa Scale (NOS) for observational studies. The NOS evaluates three domains: selection of participants (up to 4 points), comparability of study groups (up to 2 points), and ascertainment of outcomes (up to 3 points), with a maximum score of 9 points. Studies were classified as low quality (0–3 points), moderate quality (4–6 points), or high quality (7–9 points). Item-level judgments were extracted and analyzed to provide an evaluation of methodological domains. Evaluation was directed toward population selection, the accuracy of LN ascertainment, the adequacy and duration of follow-up, and the rigorousness of outcome assessment. Any disagreements in quality scoring were resolved through discussion.

### Exposure and outcomes

The primary exposure was the presence of *APOL1* HR genotypes, defined under a recessive genetic model as having two risk alleles (G1/G1, G1/G2, or G2/G2), compared with LR genotypes (G0/G0, G0/G1, or G0/G2). The G1 haplotype consists of two nonsynonymous missense variants (rs73885319 [S342G] and rs60910145 [I384M]) in near-complete linkage disequilibrium, while the G2 haplotype is characterized by a six–base pair in-frame deletion (rs71785313). The primary outcome was the development of LN-associated kidney failure. Across the included studies, this composite endpoint encompassed both treated kidney failure (defined as the requirement for maintenance hemodialysis, peritoneal dialysis, or kidney transplantation) and eGFR-defined kidney failure (variably defined in the primary studies as a sustained eGFR ≤ 15 mL/min/1.73 m^2^ or <10 mL/min/1.73 m^2^). Study-specific definitions and ascertainment methods are detailed in Table 1. Secondary outcomes included progression to advanced CKD when reported. Advanced CKD was strictly defined according to the original authors established criteria, which varied methodologically from specific eGFR thresholds (e.g., sustained eGFR <30 or <50 mL/min/1.73 m^2^) to the utilization of clinical diagnostic codes for CKD.

**Table 1:** Characteristics of the included studies.

Characteristics	Freedman	Vajgel	Chung	Blazer
	(2014) (17)	(2020) (10)	(2023) (11)	(2021) (18)
Number of patients	1,389	201	195	100
Female, %	90	89	89	99
Ethnicity	African American	Non-white Brazilian	African American	West African
Setting	United States	Brazil	United States	Ghana
*APOL1* LR, *n*	1,111	197	168	88
*APOL1* HR, *n*	278	4	27	12
Genotyping technique	TaqMan OpenArray genotyping platform (Sequenom)	TaqMan OpenArray genotyping platform (applied biosystems)	Expanded MultiEthnic genotyping array platform (illumina infinium)	TaqMan OpenArray genotyping platform (applied biosystems)
Kidney failure definitions	Treated kidney failure (receiving dialysis or having undergone kidney transplantation)	Treated kidney failure (need for renal replacement therapy) OR eGFR-defined kidney failure (eGFR <10 mL/min/1.73 m^2^)	Treated kidney failure (dependence on peritoneal dialysis, hemodialysis, and/or kidney transplantation)	Treated kidney failure (referral for hemodialysis) OR eGFR-defined kidney failure (eGFR ≤ 15 mL/min/1.73 m^2^)
Outcome ascertainment methods	Identified through rheumatology, nephrology, and kidney transplant services, CMS databases, and an i2b2 EHR query approach	Retrospective and prospective chart review tracking changes in serum creatinine/eGFR and initiation of replacement therapy	Extraction from BioVU genetic database linked to de-identified EHRs, followed by extensive manual chart review	Prospective longitudinal follow-up at 6-month intervals in an outpatient clinic using clinical referrals and lab assessments
NOS score	8/9	6/9	7/9	7/9

APOL1 LR, APOL1 Low risk variants; APOL1 HR, APOL1 High Risk variants; NOS, Newcastle-Ottawa Quality Assessment Scale; eGFR, estimated Glomerular Filtration Rate; CMS, Centers for Medicare & Medicaid Services; i2b2, Informatics for Integrating Biology and the Bedside; EHR, Electronic Health Records.

### Statistical analysis

Pooled effect estimates were calculated using the Mantel–Haenszel method based on raw 2 × 2 contingency frequencies, applying a random-effects model with the Restricted Maximum Likelihood (REML) method to account for anticipated clinical and methodological heterogeneity across studies. The Hartung-Knapp-Sidik-Jonkman (HKSJ) adjustment was implemented to provide a more robust and conservative estimation of the 95% confidence intervals due to the small number of included studies. Furthermore, a standard continuity correction was automatically applied to accommodate any study arm with zero events. Between-study heterogeneity was assessed using Cochran’s *Q* test and quantified with the *I*^2^ statistic, with values >50% indicating substantial heterogeneity. All pooled results are presented with 95% CIs, and statistical significance for the overall pooled effect was determined using the *t*-distribution as dictated by the HKSJ adjustment. Due to the limited number of included studies (*k* = 4), formal assessments of publication bias, such as funnel plot visualization and Egger’s regression test, were not performed. To ensure the robustness of our findings, a comprehensive suite of sensitivity analyses was conducted. A leave-one-out analysis was performed to evaluate the influence of individual heavily weighted cohorts. Additionally, common-effect versus random-effects models were compared, an alternative continuity correction (0.1) for zero-event data was applied, and a sensitivity analysis excluding the study with zero events in the high-risk group was conducted. Finally, a prediction interval was calculated to estimate the expected range of true effects in future clinical settings. All analyses were conducted using the *meta* package in R software (version 4.5.2; R Foundation for Statistical Computing, Vienna, Austria) (16).

## Results

### Study selection

**Figure 1:**
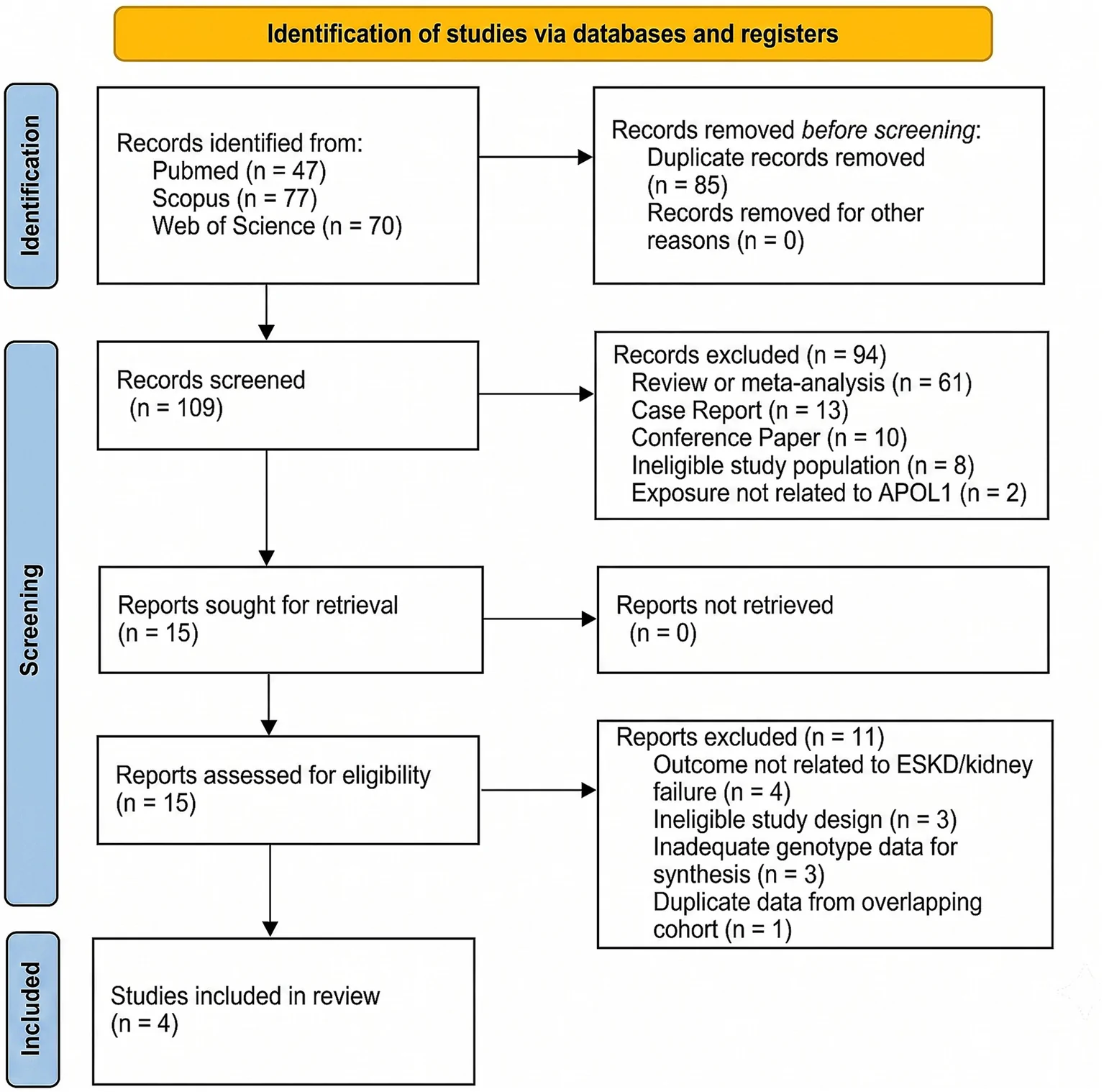
PRISMA 2020 template for the search and selection strategy used in this analysis. Review of literature according PRISMA 2020 flowsheet.

The study selection process is summarized in Figure 1. A total of 194 records were identified from PubMed (*n* = 47), Scopus (*n* = 77), and Web of Science (*n* = 70). After removing 85 duplicates, 109 records were screened by title and abstract, of which 94 were excluded. Fifteen full-text reports were assessed for eligibility. Of these, 11 were excluded due to: outcomes not related to ESKD/kidney failure (*n* = 4), ineligible study design (*n* = 3), inadequate genotype data for synthesis (*n* = 3), and overlapping cohorts (*n* = 1). Ultimately, four observational studies met all inclusion criteria and were included in the meta-analysis (Figure 1).

### Study characteristics

The characteristics of the included studies are presented in Table 1. The four included observational studies (comprising two cohort and two case–control designs) evaluated patients with LN of African ancestry. Geographically, two studies were conducted in North America (United States) (11, 17), one in South America (Brazil) (10), and one in West Africa (Ghana) (18). Across the included studies, a total of 1,885 patients of African ancestry were analyzed, with relevant sample sizes ranging from 100 to 1,389 participants per study. The proportion of female participants ranged from 89 to 99%. Overall, 321 patients carried *APOL1* HR genotypes, while 1,564 patients carried LR genotypes. Genotyping techniques varied across studies and included TaqMan OpenArray platforms and expanded multiethnic genotyping arrays.

### Risk of bias assessment

Methodological quality assessed using the Newcastle–Ottawa Scale, ranged from 6 to 8 points. As detailed in Supplementary Table S1, item-level evaluation revealed robust LN ascertainment and outcome assessment across most studies. Important methodological heterogeneity was observed in confounding control with rigorous genomic ancestry adjustments utilized by Freedman et al. (17) and Chung et al. (11), versus reliance on self-reported race in Vajgel et al. (10), and in follow-up durations, which were highly adequate in most cohorts but limited to a 12-month window in Blazer et al. (18).

### APOL1 genotypes and chronic kidney disease

Three of the included studies (Blazer et al. (18), Chung et al. (11), and Vajgel et al. (10)) reported an association between *APOL1* HR genotypes and the development or progression of CKD in patients with SLE. Although all studies describing CKD outcomes showed a positive association between *APOL1* HR genotypes and CKD-related outcomes, substantial heterogeneity in CKD definitions, ranging from specific eGFR thresholds such as <30 mL/min/1.73 m^2^ or <50 mL/min/1.73 m^2^ to the use of clinical diagnostic codes, precluded quantitative pooling of these data. Accordingly, CKD outcomes were summarized descriptively, and meta-analysis was restricted to the more uniformly defined and clinically robust endpoint of ESKD. Four studies evaluated the association between *APOL1* HR genotypes and the odds of LN-associated ESKD in patients with SLE (Supplementary Table S2). Pooled analysis using a random-effects model demonstrated that *APOL1* HR genotypes were associated with significantly increased odds of LN-associated ESKD compared with LR genotypes (pooled OR = 2.66; 95% CI, 1.63–4.32; *t*₃ = 6.39, *p* = 0.008).

### Heterogeneity and robustness of findings

**Figure 2:**
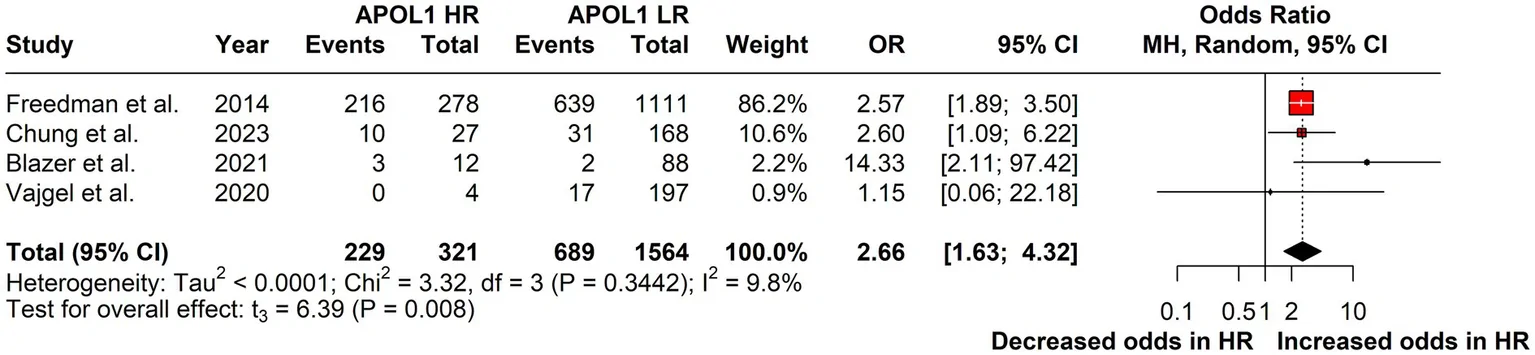
Forest plot showing the association between *APOL1* high-risk genotypes and end-stage renal disease in patients with Systemic lupus erythematosus.

While the pooled analysis yielded a statistically significant result, individual study significance varied. Specifically, the association in Vajgel et al. (10) did not reach statistical significance independently, owing to wide confidence intervals crossing the null. The calculated between-study heterogeneity was low (*I*^2^ = 9.8%; Cochran’s *Q* = 3.32, df = 3, *p* = 0.34) (Figure 2).

Robustness and sensitivity analyses revealed that while the pooled estimate is heavily influenced by the largest cohort, the direction of the effect remains consistent. The leave-one-out analysis demonstrated that the statistical significance of the pooled odds ratio is lost when omitting either Freedman et al. (17) or Chung et al. (11), with confidence intervals crossing the null value (Supplementary Figure S1). However, the association remained stable and statistically significant when comparing random-effects to common-effect models, applying alternative continuity corrections, and excluding the zero-event study (Supplementary Table S3, Supplementary Figure S2). Furthermore, the calculated 95% prediction interval (1.67–4.22) reflected a consistently increased odds across varied clinical settings (Supplementary Table S3).

## Discussion

In this systematic review and meta-analysis, we demonstrate that *APOL1* HR genotypes are associated with substantially increased odds of LN-associated ESKD in patients with SLE. The pooled estimate indicates that carriers of two *APOL1* risk alleles have 2.66-fold higher odds of LN-associated ESKD compared with individuals with LR genotypes. This finding provides quantitative confirmation of observations previously reported in individual studies of SLE patients, particularly among populations of African descent and admixed cohorts with African genetic heritage, and establishes the magnitude of the association with *APOL1* in this specific clinical context (6, 13, 19).

The high proportion of females (89–99%) observed uniformly across all included studies reflects the well-established disproportionate burden of SLE on women (11, 17, 18, 20). While male sex is a recognized clinical risk factor for more severe renal involvement and accelerated progression to kidney failure in SLE, our pooled studies predominantly consisted of female patients, which aligns with the global epidemiological landscape of the disease but limits our ability to perform sex-stratified outcome analyses (21, 22).

**Figure 3:**
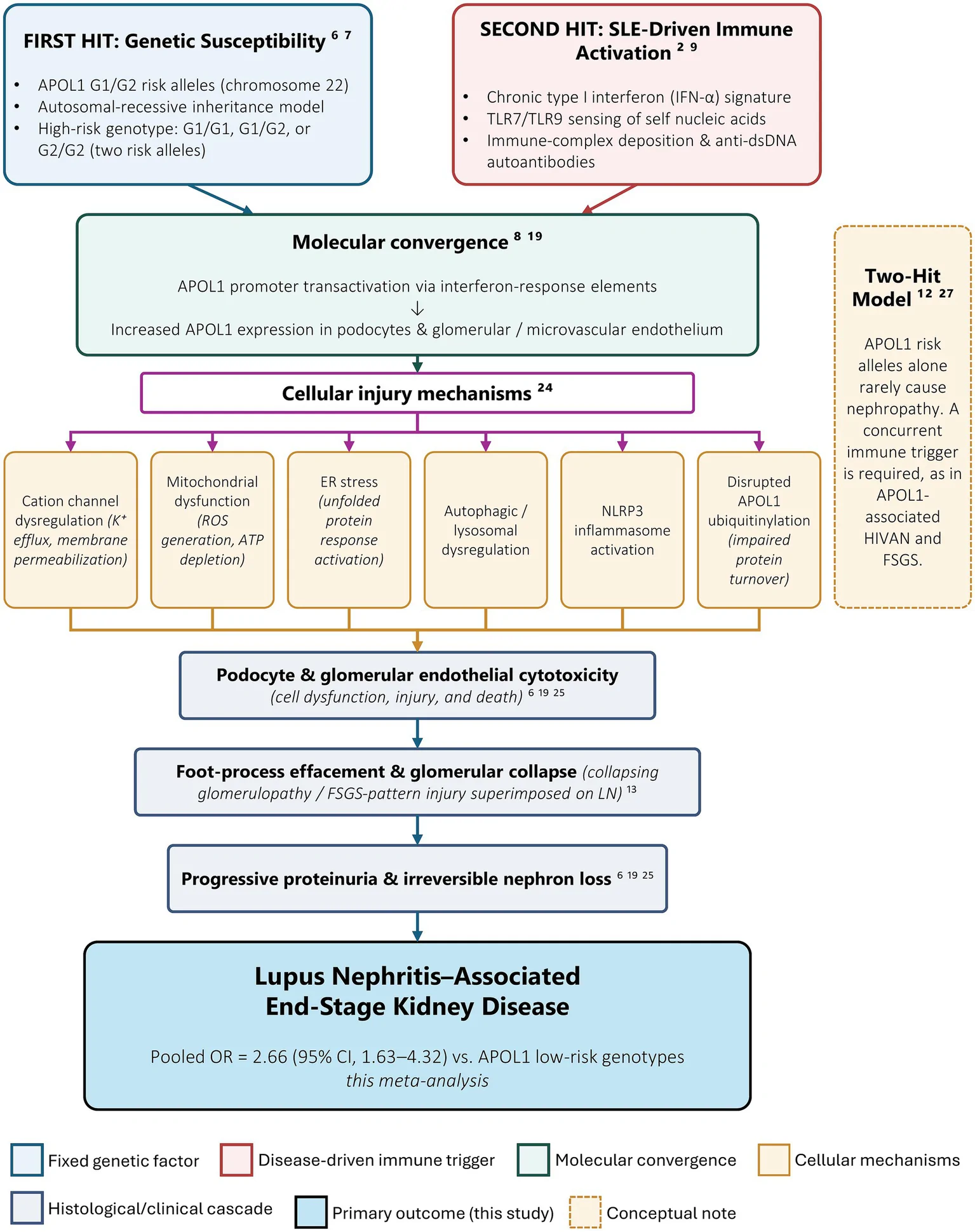
Proposed two-hit mechanistic model linking *APOL1* high-risk genotypes to Lupus nephritis (LN)-associated kidney failure. Genetic susceptibility (blue, first hit) combines with SLE-driven immune activation (red, second hit) to transactivate the *APOL1* promoter and increase *APOL1* expression in podocytes and glomerular endothelium (teal), precipitating the cellular injury mechanisms shown (amber) and culminating in podocyte/endothelial cytotoxicity, glomerular collapse, and progressive nephron loss (slate blue), which manifest as LN-associated end-stage kidney disease (navy)—The primary outcome of this meta-analysis (pooled OR = 2.66; 95% CI, 1.63–4.32). The dashed inset summarizes the two-hit hypothesis. APOL1, apolipoprotein L1; ER, endoplasmic reticulum; FSGS, focal segmental glomerulosclerosis; HIVAN, HIV-associated nephropathy; IFN, interferon; LN, Lupus nephritis; NLRP3, NOD-like receptor family pyrin domain-containing 3; ROS, reactive oxygen species; TLR, toll-like receptor.

The direction of the association between *APOL1* HR genotypes and ESKD was consistent across all included studies, with no study reporting an inverse association. Although statistical significance was not reached in every individual cohort (e.g., Vajgel et al. (10)), between-study heterogeneity was remarkably low (*I*^2^ = 9.8%), suggesting that differences in study size, geographic setting, and genotyping platforms did not substantially influence the direction or strength of the pooled effect. This consistency strengthens confidence in *APOL1* as a robust prognostic marker for adverse renal outcomes in LN, despite the limited number of available studies (11, 19, 23). The strong association observed in LN is biologically plausible and supported by extensive mechanistic evidence. *APOL1* expression is upregulated by interferon signaling pathways, which are chronically activated in SLE (7, 24). Experimental studies have shown that *APOL1* risk variants exert cytotoxic effects on podocytes and endothelial cells, particularly under inflammatory conditions, leading to glomerular collapse and progressive nephron loss (6, 19, 25). These findings support the concept that LN represents an immunologically permissive environment in which APOL1-mediated kidney injury is amplified (Figure 3).

The magnitude of the association observed with *APOL1* HR genotypes in SLE exceeds that reported in unselected CKD populations, where effect estimates are generally modest (26). Our pooled estimate (OR = 2.66) further demonstrates that while both LN and HIV-associated nephropathy (HIVAN) share a common pathophysiological “*second hit”* driven by intense immune activation, the magnitude of the association in LN is notably more modest than the exceptionally high associations classically reported in HIVAN (12–14). This pattern supports the *second-hit* hypothesis, whereby APOL1-mediated nephropathy manifests most aggressively in the presence of immune activation, viral infection, or sustained inflammatory stress (6, 27).

Previous systematic reviews and meta-analyses have evaluated the association between *APOL1* genotypes and kidney disease outcomes in heterogeneous populations, including individuals with focal segmental glomerulosclerosis and CKD of various etiologies (23, 24). However, these studies did not focus specifically on LN, nor did they provide disease-specific effect estimates for ESKD in this population. By restricting our analysis to LN studies and employing a recessive genetic model, our study addresses this important gap and offers clinically relevant risk estimates tailored to a high-risk subgroup. Importantly, while the present analysis quantifies the odds of LN-associated ESKD, the impact of *APOL1* HR genotypes on the initial incidence of LN among patients with SLE has historically been a subject of debate. However, recent evidence from a case–control study by Patel et al. demonstrates that *APOL1* high-risk genotypes are indeed associated with significantly higher odds of developing incident LN (OR 3.27) (28). Current mechanistic models suggest that *APOL1* variants primarily act as accelerators of kidney function decline following an inflammatory “*second hit,”* rather than initiating the autoimmune glomerular injury itself (19, 23). Whether these risk alleles inherently lower the threshold for developing incident LN in an otherwise unaffected SLE patient requires further elucidation. Future large-scale, prospective studies evaluating SLE cohorts without baseline kidney involvement are needed to definitively determine the role of *APOL1* in LN susceptibility.

Furthermore, the precise allelic dose required to confer risk in LN remains an area of active investigation. We acknowledge that emerging evidence in other nephropathies suggests that individuals heterozygous for *APOL1* risk variants carrying a single risk allele (G0/G1 or G0/G2) may exhibit an intermediate risk or distinct clinical phenotypes compared with those carrying zero risk alleles (G0/G0) (29, 30). Because the primary LN studies currently available group patients with zero and one risk allele into a single reference category, the specific prognostic impact of a single risk allele could not be quantified in this meta-analysis. This represents a critical gap in the current literature that warrants granular exploration in future longitudinal studies to further refine genetic risk stratification (30).

The findings of this meta-analysis suggest that *APOL1* high-risk status may serve as a valuable genetic marker for identifying SLE patients with increased odds of LN-associated ESKD. Given the heterogeneity of LN clinical course and response to therapy, incorporation of genetic risk factors such as *APOL1* may enhance existing prognostic models and inform the intensity of monitoring in patients of African ancestry, particularly in interferon-driven disease states (7, 14, 19). Although routine *APOL1* genotyping is not yet standard of care in LN, accumulating evidence supports its potential role in precision nephrology and highlights it as a potential target for future therapeutic interventions (19, 27). However, the clinical translation of routine *APOL1* genetic testing introduces profound ethical, psychosocial, and systemic complexities. In populations of African descent, the identification of HR genotypes carries the potential for genetic stigmatization and may inadvertently exacerbate existing healthcare disparities (31). Furthermore, predictive genetic testing raises substantial concerns regarding insurability, particularly the risk of life and health insurance discrimination based on the probability of future kidney failure (31, 32). The psychological burden placed on asymptomatic individuals or those with early-stage LN who are identified as high-risk necessitates the development of comprehensive genetic counseling frameworks (31). Therefore, while *APOL1* risk stratification holds immense clinical promise, its integration into standard rheumatological and nephrological care must be accompanied by robust ethical guidelines, protective socioeconomic policies, and culturally sensitive patient education (31, 33).

Several limitations should be acknowledged. First, the quantitative synthesis relies on only four studies, with the pooled estimate predominantly driven by a single large cohort (Freedman et al. (17), 86.2% weight). While the directional effect was consistently positive, this predominance affects the precision of the pooled estimate. Second, the included cohorts predominantly consist of African American individuals from North America, limiting generalizability to the broader African diaspora where differing socio-environmental factors and genetic admixtures may uniquely influence renal outcomes. Third, the reliance on retrospective and electronic health record-based designs introduces potential case ascertainment bias. Fourth, severe reporting heterogeneity in covariate structures (e.g., genomic ancestry versus self-reported race) and zero-event cells precluded the pooling of adjusted effect estimates. Consequently, our crude estimates do not account for critical confounders such as age, sex, and immunosuppressive treatment; however, it is reassuring that the largest primary studies independently maintained a robust association even after stringent genomic adjustment. Lastly, the small number of studies precluded formal subgroup meta-analyses by study design, although our leave-one-out analysis effectively addressed this by modeling the exclusion of the sole case–control cohort. Future prospective studies are needed to evaluate the integration of *APOL1* genotyping into clinical decision-making frameworks, to explore interactions with immunosuppressive therapies, and to assess whether emerging APOL1-targeted interventions may modify renal outcomes in SLE (11, 23).

In conclusion, pooled analyses demonstrate that *APOL1* HR genotypes are associated with substantially increased odds of LN-associated ESKD in patients with SLE. This systematic review and meta-analysis provide quantitative evidence supporting *APOL1* as a clinically relevant genetic modifier of renal prognosis in SLE, reinforcing its potential role in risk stratification and future precision medicine approaches in this high-risk population.

## Data Availability

The original contributions presented in the study are included in the article/Supplementary material, further inquiries can be directed to the corresponding author.
