# Supplementary material for: Apolipoprotein L1 risk genotypes and odds of lupus nephritis-associated kidney failure in systemic lupus erythematosus: a systematic review and meta-analysis
Source: Front Med (Lausanne). 2026 Aug 19;13:1903888. doi: 10.3389/fmed.2026.1903888 (PMC13534799; doi:10.3389/fmed.2026.1903888)
Supplement: Supplementary file 1 [file Supplementary_file_1.docx]

**Supplementary Materials for**

**Apolipoprotein L1 Risk Genotypes and Odds of Lupus Nephritis-Associated Kidney Failure in Systemic Lupus Erythematosus: A Systematic Review and Meta-Analysis**

**Authors**

Silvia E. Aldana-Pérez ¹, Alex Domínguez Vargas ²^,^ ³, Gustavo Aroca-Martinez¹^,^ ⁴, Ana Moreno-Woo³, Luis Fang³, Gloria Garavito De Egea³, Eduardo Egea Bermejo³, Henry J. González-Torres ¹^,^ ²

**Affiliations**

¹ Centro de Investigaciones en Ciencias de la Vida, Facultad de Ciencias de la Salud, Universidad Simón Bolívar, Barranquilla 080001, Colombia

² Data Analysis and Mining Department, D&P Consulting Service SAS, Barranquilla 080001, Colombia

³ División Ciencias de la Salud, Universidad del Norte, Barranquilla 080001, Colombia

⁴ Departamento de Medicina Interna, Clínica de la Costa, Barranquilla 080001, Colombia

**This file includes:**

**Supplementary Method S1: Complete Search Strategies**

**Supplementary Table S1: Item-Level Risk of Bias Assessment using the Newcastle-Ottawa Scale**

**Supplementary Table S2: Raw 2x2 contingency data extracted for the quantitative synthesis of lupus nephritis-associated end-stage renal disease.**

**Supplementary Table S3. Sensitivity and Robustness Analyses of the Association Between APOL1 High-Risk Genotypes and Lupus Nephritis-Associated ESRD**

**Supplementary Figure S1: Leave-one-out sensitivity analysis for the association between *APOL1* high-risk genotypes and lupus nephritis-associated end-stage renal disease.**

**Supplementary Figure S2: Forest plot comparing common-effect and random-effects models for the association between *APOL1* high-risk genotypes and lupus nephritis-associated end-stage renal disease.**

**Supplementary Method 1: Complete Search Strategies**

**PubMed (MEDLINE)**

("Lupus Nephritis"[Mesh] OR "Lupus Erythematosus, Systemic"[Mesh] OR "lupus nephritis"[tiab] OR "LN"[tiab] OR "systemic lupus erythematosus"[tiab] OR "SLE"[tiab]) AND ("Apolipoprotein L1"[Mesh] OR "APOL1"[tiab] OR "apolipoprotein L1"[tiab])

**Web of Science**

("lupus nephritis" OR "LN" OR "systemic lupus erythematosus" OR "SLE") AND ("APOL1" OR "apolipoprotein L1")

**Scopus**

("lupus nephritis" OR "LN" OR "systemic lupus erythematosus" OR "SLE") AND ("APOL1" OR "apolipoprotein L1")

**Supplementary Table S1: Item-Level Risk of Bias Assessment using the Newcastle-Ottawa Scale**

| **Methodological**  **Domain** | **Freedman et al.  (2014)** | **Vajgel et al.  (2020)** | **Chung et al.  (2023)** | **Blazer et al.  (2021)** |
| --- | --- | --- | --- | --- |
| **Study Design** | Case-Control | Cohort | Cohort | Cohort |
| **SELECTION  (Max 4★)** | **4★** | **3★** | **3★** | **3★** |
| Population Selection Representativeness | 1★  Multi-center, population-based African American sample | 1★  Multi-center, non-white Brazilian LN outpatients | 1★  BioVU genetic biobank linked to EHRs | 1★  Consecutive outpatient sample in Ghana |
| Selection of Controls   Non-exposed | 1★  Community SLE controls without nephropathy | 1★  Healthy blood donors from the same region | 1★  Internal EHR SLE controls | 1★  Drawn from the same source population |
| LN  Exposure Ascertainment | 1★  Strict clinical criteria and renal biopsy reports available for 66.8% | 1★  Gold standard: 100% biopsy-proven LN ascertainment | 0★  Algorithms/EHR billing codes; inherent misclassification risk | 1★  Standardized prospective clinical and lab metrics |
| Outcome not present at start /  Case definition | 1★  Appropriate LN-ESRD vs SLE non-nephropathy case-control definition | 0★  Retrospective ascertainment limits confirmation of true baseline status | 1★  Longitudinal tracking from index SLE diagnosis | 0★  53% had LN at baseline; mixed baseline status |
| **COMPARABILITY  (Max 2★)** | **2★** | **0★** | **2★** | **2★** |
| Ancestry Adjustment & Confounding Control | 2★  Rigorously adjusted for genetic admixture proportions using LD-pruned SNPs, plus age and sex | 0★  Relied on self-reported skin color; lacked DNA for genetic ancestry informative markers [AIMs] | 2★  Genomic principal components used to define and adjust for ancestry, plus age and sex | 2★  100% West African homogenous cohort eliminates major admixture bias; adjusted for SLEDAI and disease duration |
| **OUTCOME / EXPOSURE  (Max 3★)** | **2★** | **3★** | **2★** | **2★** |
| Outcome Assessment | 1★  Standardized medical records and registries | 1★  Standardized medical records tracking eGFR and RRT | 1★  PheWAS validated by extensive manual investigator chart review for ESRD | 1★  Standardized prospective clinical and lab metrics |
| Follow-up length  adequacy | N/A  Case-control design relies on exposure ascertainment | 1★  Median 78–89 months; highly adequate for CKD/ESRD progression | 1★  Median 8.7 years; adequate for LN-ESRD | 0★  12 months follow-up; critically short for natural ESKD progression |
| Adequacy of follow-up  non-response rate | 1★  Same non-response/missingness rate for both groups | 1★  Complete clinical follow-up for the analyzed cohort | 0★ EHR inherent missingness / loss to follow-up unspecified | 1★  Prospective tracking with documented attrition |
| **TOTAL NOS SCORE** | **8 / 9** | **6 / 9** | **7 / 9** | **7 / 9** |
| CKD: Chronic Kidney Disease; LN: Lupus Nephritis; EHR: Electronic Health Record; SLE: Systemic Lupus Erythematosus | | | | |

**Supplementary Table S2: Raw 2x2 contingency data extracted for the quantitative synthesis of lupus nephritis-associated end-stage renal disease.**

|  |  | **APOL HR** | | **APOL LR** | |
| --- | --- | --- | --- | --- | --- |
| **Author** | **Year** | **ESRD Event** | **Total (n)** | **ESRD Event** | **Total (n)** |
| Freedman et al. | 2014 | 216 | 278 | 639 | 1111 |
| Vajgel et al. | 2020 | 0 | 4 | 17 | 197 |
| Chung et al. | 2023 | 10 | 27 | 31 | 168 |
| Blazer et al. | 2021 | 3 | 12 | 2 | 88 |
| HR: APOL1 high-risk genotype (two APOL1 risk alleles); LR: APOL1 low-risk genotype (zero or one APOL1 risk allele); ESKD: End-stage kidney disease. | | | | | |

**Supplementary Table S3. Sensitivity and Robustness Analyses of the Association Between APOL1 High-Risk Genotypes and Lupus Nephritis-Associated end-stage renal disease.**

| **Sensitivity Analysis**  **Model** | **k** | **Pooled OR** | **95% CI** | **p-value** | **I² (%)** |
| --- | --- | --- | --- | --- | --- |
| Primary Analysis  (Random-effects, REML) | 4 | 2.66 | 1.63 – 4.32 | 0.008 | 9.8% |
| Common (Fixed)  Effect Model (MH) | 4 | 2.62 | 1.97 – 3.49 | < 0.001 | 9.8% |
| Alternative Continuity  Correction * | 4 | 2.66 | 1.63 – 4.32 | 0.008 | 9.8% |
| Exclusion of Zero-Event  Study ** | 3 | 2.68 | 1.24 – 5.78 | 0.032 | 33.6% |
| Prediction Interval *** | 4 | 2.66 | 1.67 – 4.22 | - | - |
| k, number of studies; OR: odds ratio; CI: confidence interval; REML: restricted maximum likelihood; MH: Mantel-Haenszel. | | | | | |
| * Application of alternative continuity configurations did not significantly alter the Mantel-Haenszel pooled estimate | | | | | |
| ** Excludes Vajgel et al. to assess the impact of zero-cell frequency correction on the pooled variance. | | | | | |
| *** Represents the 95% Prediction Interval for the expected true effect in future clinical settings. | | | | | |

**Supplementary Figure S1: Leave-one-out sensitivity analysis for the association between *APOL1* high-risk genotypes and lupus nephritis-associated end-stage renal disease.**

**Supplementary Figure S2: Forest plot comparing common-effect and random-effects models for the association between *APOL1* high-risk genotypes and lupus nephritis-associated end-stage renal disease.**
